# Bayesian Estimation of Sensitivity and Specificity of Rose Bengal, Complement Fixation, and Indirect ELISA Tests for the Diagnosis of Bovine Brucellosis in Ethiopia

**DOI:** 10.1155/2016/8032753

**Published:** 2016-08-09

**Authors:** T. Getachew, G. Getachew, G. Sintayehu, M. Getenet, A. Fasil

**Affiliations:** National Animal Health Diagnostic and Investigation Center (NAHDIC), Ethiopian Ministry of Livestock and Fisheries Development, P.O. Box 04, Sebeta, Ethiopia

## Abstract

Test evaluation in the absence of a gold standard test was conducted for the diagnosis and screening of bovine brucellosis using three commercially available tests including RBPT, CFT, and I-ELISA in National Animal Health Diagnostic and Investigation Center (NAHDIC) Ethiopia. A total of 278 sera samples from five dairy herds were collected and tested. Each serum sample was subjected to the three tests and the results obtained were recorded and the test outcomes were cross-classified to estimate the sensitivity and specificity of the tests using Bayesian model. Prior information generated on the sensitivity and specificity of bovine brucellosis from published data was used in the model. The three test-one population Bayesian model was modified and applied using WinBug software with the assumption that the dairy herds have similar management system and unknown disease status. The Bayesian posterior estimate for sensitivity was 89.6 (95% PI: 79.9–95.8), 96.8 (95% PI: 92.3–99.1), and 94 (95% PI: 87.8–97.5) and for specificity was 84.5 (95% PI: 68–94.98), 96.3 (95% PI: 91.7–98.8), and 88.5 (95% PI: 81–93.8) for RBT, I-ELISA, and CFT, respectively. In this study I-ELISA was found with the best sensitivity and specificity estimates 96.8 (95% PI: 92.3–99.1) and 96.3 (95% PI: 91.7–98.8), compared to both CFT and RBPT.

## 1. Introduction 

Brucellae are Gram-negative, facultative intracellular bacteria that can infect many species of animals and man. Ten species are recognized within the genus* Brucella*. There are 6 “classical” species*: B. abortus, B. melitensis, B. suis, B. ovis, B. canis*, and* B. neotomae* [1, 2] and, more recently, other four species have been recognized [3]. The principal manifestations of brucellosis are reproductive failure such as abortion or birth of unthrifty newborn and infertility [4, 5]. Brucellosis in animals and humans is still common in the Middle East, Asia, Africa, South and Central America, the Mediterranean Basin, and the Caribbean.* Brucella melitensis* is particularly common in the Mediterranean basin and it has also been reported in Africa, India, and Mexico [6].

Previous studies carried out in Ethiopia on bovine brucellosis using Rose Bengal and complement fixation tests described higher prevalence in intensive and semi-intensive dairy farms than extensive farms [1, 7, 8]. In 1987, the World Organization for Animal Health reported 20% prevalence of brucellosis, being higher around large towns than in rural areas [9]. In central highlands of Ethiopia, 4.2% prevalence of brucellosis was reported in zebu cattle [7]. Eshetu et al. [10] reported a prevalence of 10% in smallholder farms of central Ethiopia (Wuchale-Jida district) near Addis Ababa in 2005. Kebede et al. [11] reported a prevalence of 11% in cattle under extensive management systems. Studies conducted in different regions in 2003 and 2005 have reported animal level prevalence of 0.8% and 3.2% and herd prevalence of 2.9% and 42.3% [8, 12]. Another study in Ethiopia from 2003 to 2004 has reported a prevalence of 1.6% and a herd level prevalence of 13.7% [1]. A more recent study from 2011 to 2012 on exotic and crossbred dairy cattle and breeding farms has reported animal level prevalence of 1.9% and herd level prevalence of 10.6% in Ethiopia [13].

Serological tests are widely used to conduct several epidemiological studies and diagnostic purposes, but there is no perfect serological test [14, 15]. However, the diagnostic performance and discriminative ability of a test could be evaluated by comparing the sensitivity and specificity of several tests analytically [14, 16]. The diagnostic performance of a test could be evaluated by comparison with standard reference test and analyzed using latent models [17–19]. The objective of this studywas to evaluate diagnostic performance and discriminative ability of Rose Bengal Plate Test (RBPT), complement fixation test (CFT), and indirect enzyme linked immunosorbent assay (I-ELISA) tests used for screening and confirmatory diagnosis of bovine brucellosis in Ethiopia using Bayesian method. This study is one of a kind in the context of field diagnostic test evaluation for bovine brucellosis in Ethiopia which has significant importance for disease surveillance and future control endeavors.

## 2. Materials and Methods

### 2.1. Study Area and Population

The study was conducted in five dairy farms, namely, Sululta, Awash, Wonji, Adami Tulu, and Alage located at 35, 100, 90, 170, and 200 km from Addis Ababa, respectively. The management system and breed of the farms were similar and the disease status was unknown. Thus, the farms were assumed as one population. All animals aged above six months in the farm were included in the sampling and the total number of the study animals was 278 pure and crossbreed Holstein Frisian dairy cows.

The farm history during sampling showed that there was no vaccination against brucellosis in all farms. Blood samples of 5–7 mL were collected in plain vacutainer tube from the jugular vein. The samples were allowed to clot for 2-3 h at room temperature. Then the serum was extracted by spinning at 2500 rpm for five minutes and kept in refrigerator at −20°C until the test is conducted. All farms except Sululta are located in the Great Rift Valley area of Ethiopia.

### 2.2. Diagnostic Tests

All serological tests conducted for test evaluation were performed at NAHDIC, Sebeta, Ethiopia (Bacterial Serology Laboratory).

#### 2.2.1. Rose Bengal Test

 Rose Bengal Test was conducted following the procedure described by OIE 2009. Antigen for the Rose Bengal Test was prepared from* B. abortus* strain 99 stained with Rose Bengal dye and suspended in acid buffer pH 3.65. Equal volume (30 *μ*L) of antigen and test serum is brought together using a micropipette channel; then after thorough mixing it was rocked for four minutes; finally the result was read using magnifying glass and recorded as positive or negative based on the absence or presence of agglutination due to antigen-antibody reaction in the serum. Rose Bengal antigen was purchased from Lillidale Diagnostics, UK.

#### 2.2.2. Complement Fixation Test

 Complement fixation test was conducted using Alton et al. [20] Method. As a principle, if a specific antibody against bovine* Brucella* is present in the serum, then antigen-antibody complex is formed and the complement will bind. The positive result of the test was when no hemolysis of the sheep RBC occurs. If there is no specific antibody against* Brucella*, the free complement exists which will cause sensitization of sheep RBC and lead to hemolysis. The validation of the result was done using positive and negative controls. Result interpretation based on the titration scale considered strong reaction when more than 75% fixation of the complement (3+) occurred at a dilution of 1 : 5 and the reaction was classified as weak positive with 50% fixation of complement (2+) that occurred at a dilution of 1 : 10 and above.* Brucella *antigen for the complement fixation test was prepared from* B. abortus* S99 and standardized against the OIEISS to give 50% fixation at a dilution of 1/200.* Brucella* antigen and positive control for complement fixation test were obtained from AH-VLA (Animal Health Veterinary Laboratory Agency), UK. Hemolytic serum and guinea pig complement was obtained from ID VET (Innovative Veterinary Diagnostic) Company.

#### 2.2.3. Indirect ELISA Test

Test was performed according to the manufacturer's instructions and procedures. Indirect ELISA kit obtained from VLA Lillidale Animal Health Limited, Badbury View, Bothenwood, Wimborne, Dorset BH214HU, UK (https://www.gov.uk/government/organisations/animal-health-and-veterinary-laboratories-agency).

*Reagent Preparation*. The dilution buffer was prepared by adding 5 tablets PBS 0.5 mL phenol red indicator and 250 *μ*L of Tween 20 to 500 mL distilled water; the pH was adjusted to 7.2. Then solution was prepared by adding the contents of the ampoule of Na_2_HPO_4_ and 1 mL of Tween 20 to 10 liters of distilled water. The substrate buffer prepared was by dissolving 1 tablet in 120 mL distilled water. The chromogen was prepared by dissolving 2 tablets in 1 mL of sterile distilled water. The stopping solution was prepared by diluting the ampoule of sodium azide with 500 mL of distilled water. Antigen was prepared from approved smooth lipopolysaccharides* B. abortus* strain 99 1 *µ*g/m/L coated in 0.05 M carbonate/bicarbonate buffer, pH 9.6 onto flat bottom microplate wells. Positive and negative controls were reconstituted with 1 mL sterile distilled water and allowed until an even suspension is obtained before use. The test procedure, first a 1/40 predilution of all tests and control sera was made; then the plate was prepared by adding 80 *μ*L of diluting buffer to wells followed by transferring of 20 *μ*L of prediluted samples into a 96-well microplate coated with* Brucella* lipopolysaccharides (LPS). The optical density (OD0) was set at 405 nanometers blanked on well H12 and the presence or absence of antibodies against LPS of* Brucella* was determined by comparing the mean OD of positive controls. Color development within a well indicates that the sample has antibodies to* Brucella*. The validation criteria are as follows  the cut-off value for positive/negative was calculated as 10% of the mean OD of positive control wells. Any test sample giving an OD equal to or above this value should be considered positive.

#### 2.2.4. Test Evaluation Using Bayesian Model

Estimation of diagnostic test sensitivity and specificity through Bayesian modeling has an advantage to provide more stable point and interval estimates without the necessity of large sample sizes [21, 22]. One of the reasons why Bayesian approach was employed was that it can give good estimates of sensitivity and specificity in the absence of gold standard method like culture and isolation. The Bayesian approach is a well-established methodology for robust diagnostic test evaluation. We could not culture samples for bacterial isolation of* Brucella* in our laboratory because of biorisk and biosecurity concern. Finally, we consider that this does not affect the results of our study.

The sensitivity and specificity of the three tests were evaluated using a total of 278 sera samples collected from five dairy farms. Each serum sample was subjected to the three tests and the results were entered into the computer. The observed data of the three tests' results was summarized in cross tabulation. Bayesian model without gold standard was applied to estimate the sensitivity and specificity estimates. Prior information for the unknown data in the model was used from published data on bovine brucellosis [15, 23].

Gall and Nielsen [15] reviewed over 50 publications in which sensitivity and specificity values of assays used for the detection of exposure to* Brucella abortus* where the sum of sensitivity and specificity values for each test was averaged to give a performance index. Similarly, comparison was made of sensitivity and specificity of I-ELISA RBT that thus we used as prior information for our data analysis.

The uncertainty of an average sensitivity and specificity obtained from the published data was transformed to the beta distribution using Betabuster free software (http://www.epi.ucdavis.edu/diagnostictests [22]). The prior information for sensitivity of RBP, I-ELISA, and CFT was of modes 0.91, 0.97, and 0.94, respectively, and the transformed beta (a, b) was beta (49.4, 6.0); (103.2, 3.73); and (89.27, 6.14), respectively. Prior mode for specificity of RBP, I-ELISA, and CFT was 0.86, 0.97, and 0.89, respectively, and the transformed beta distribution (a, b) was (22.76, 4.43); (102.1, 4.23); and (83.05, 11.14), respectively (Table 1). 

The Bayesian model for one population-three tests was modified and applied for the data using WinBUGS free software. The median value of the posterior distributions was built after 50,000 iterations and the burnout of the initial 5,000 iterations. The model sensitivity was checked using kernel density and autocorrelation graphs that showed the posterior distribution fit fairly to the data. Conditional dependence of the tests was also checked because the tests are based on similar biological basis which might lead to correlated errors leading to incorrect estimation of sensitivity and specificity [22]. Then, conditional independent Bayesian model was applied which allowed us to estimate the conditional correlations (rhoD and rhoDc) for Se and Sp, respectively, for the three tests.

## 3. Results

All sera samples were tested blindly by all the three tests (RBT, I-ELISA, and CF) independently. The tests result showed 5/278; 8/278 and 2/278 positive for RBT, I-ELISA, and CF, respectively; this indicated that I-ELISA is superior in sensitivity and specificity, followed by CF. Kappa test of the three tests showed moderate agreement (kappa = 0.70); the rhoD and rhoDc values were small and clustered around zero which indicates that the tests are conditionally independent (Table 2). 

The posterior inference for the true sensitivity of RBT, I-ELISA, and CFT was 89.6 (95% PI: 79.9–95.8), 96.8 (95% PI: 92.3–99.1), and 94 (95% PI: 87.8–97.5) and true specificity was 84.5 (95% PI: 68–94.98), 96.3 (95% PI: 91.7–98.8), and 88.5 (81–93.8), respectively. In this study, the true sensitivity and specificity of I-ELISA (96.8 95% PI (92.3–99.1) and 96.3 95% PI (91.7–98.8), resp.) were found higher than RBPT and CFT. The seroprevalence of brucellosis in these farms was estimated to be 4 (95% PI: 0.8–11.45).

The conditional correlation to evaluate conditional dependence of the three tests showed that the value estimate for both rhoD (for sensitivity) and rhoDc (for specificity) was small with the probability interval clustering around zero which showed the tests were conditionally independent. The sensitivity analysis based on the posterior distribution kernel density and autocorrelation graphs showed that the observed data fairly fit the model and prior information has not significantly influenced the median estimate (Table 3). 

## 4. Discussion

Screening and confirmatory diagnostic tests are the primary tools for successful epidemiological study. In Ethiopia, although many papers were published to determine the prevalence of bovine brucellosis in different farm settings, we could not find any published data on sensitivity and specificity of the serological tests. The knowledge on the diagnostic sensitivity and specificity of a test would help to limit diagnostic errors in classifying infected and noninfected animals correctly and to prevent excessive economical losses when the animals are wrongly classified by the tests [24].

No single serological test is appropriate in all epidemiological situations and all animal species; all tests have limitations especially when screening individual animals. Consideration should be given to all factors that impact on the relevance of the test method and test results to a specific diagnostic interpretation or application. Antigen for the Rose Bengal Test was prepared by depositing killed* B. abortus* strain 99 (Weybridge) cells stained with Rose Bengal dye and suspended in acid buffer pH 3.65. Antigen for complement fixation test was prepared from* B. abortus* strain 99 (Weybridge) and standardized against the OIEISS to give 50% fixation at a dilution of 1/200. The same* B. abortus* strain 99 (Weybridge) was also used as a source of soluble antigen extracts (smooth lipopolysaccharide (S-LPS) for the indirect ELISA). Therefore, antigen for indirect ELISA was prepared from approved smooth lipopolysaccharides* B. abortus* strain 99 1 *μ*g/m/L coated in 0.05 M carbonate/bicarbonate buffer, pH 9.6, onto flat bottom microplate wells. All the three antigens are used to detect infections due to smooth* Brucella* species as per information obtained from the manufacturer. All diagnostic kit components (i.e., antigen, reference sera, and complements) used for the test evaluation purpose were of highest quality obtained from VLA, UK, internationally recognized diagnostic kit supplier with good manufacturing practice.

Estimation of diagnostic sensitivity and specificity of a test requires knowledge of the true disease status of the animals on which the test is to be applied using the gold standard test; however, in the absence of such a gold standard test a Bayesian approach is a useful tool to evaluate the characteristics of the tests [18, 19, 25].

Bayesian method has an advantage as it provides a stable point and interval estimates without the necessity of large sample size [21, 26]. It is widely accepted that screening tests should have a higher sensitivity but could have a lower specificity. The sensitivity of RBT in the current study was fairly high (89.6 (95% PI: 79.9–95.8)) which was higher than the previous finding by Sanogo et al. (2013) [27] (54.9% (cr 23.5–95.1)).

Previous studies suggested that CFT is an appropriate confirmatory test with high specificity [16] but this was not consistent with the current finding that the specificity of CFT was moderate (88.5 (95% PI: 81–93.8)) which might be due to small population size in our study. However, Gall and Nielsen (2004) [15] reported the sensitivity and specificity of CFT as Se 81.2 and Sp 83.5, respectively, which is in agreement with our current finding. The I-ELISA was found to be the best sensitive and specific test (95% PI: 92.3–99.1 and 95% PI: 91.7–98.8, resp.) for bovine brucellosis compared to both CFT and RBPT. The possible reason for this high accuracy might be due to the fact that I-ELISA detects all isotopes of immunoglobulin IgG while CFT cannot detect them [14]. The mean sensitivity and specificity for indirect ELISA were reported as Se 96.0 and Sp 93.8 by Gall and Nielsen (2004) which was in agreement with our estimates.

The conditional dependence of the tests is that the conditional correlation rhoD and rhoDc values for sensitivity and specificity, respectively, were small and clustered around zero which indicates that the tests are conditionally independent and could be an advantage while using in test combinations [28]. The sensitivity analysis using different prior information showed that the posterior distribution kernel density and autocorrelation graphs showed that the observed data fairly fit the model and prior information has not significantly influenced the median estimates.

## 5. Conclusion and Recommendation 

Based on this observation I-ELISA had the best performance followed by CFT and RBPT in descending order of accuracy. However, the decision for the choice of diagnostic test for different purposes not only does rely on the accuracy, but also should take into consideration the capacity for the test throughput, technical complexity, and cost effectiveness. Regardless of its lower sensitivity, RBT remains the most widely used screening test because of its rapid result and cost effectiveness. Therefore, conducting test verification is very essential to know the test characteristics and to determine the type of test we require to use for the study purpose, epidemiological surveillance, or international trade. We recommend further studies should be conducted on the performance of these tests in the field setting for the diagnosis of sheep and goat brucellosis to generate sufficient information.

## Figures and Tables

**Table 1:** Prior information used for sensitivity and specificity of RBT, I-ELISA, and CFT.

	RBT	I-ELISA	CFT	
Se	81.2 (66.4–96)	96 (90.2–99.8)	89 (81.3–96.7)	Gall and Nielsen (2004) [15]
Sp	86.3 (71.64–99)	93.8 (88–99.6)	83.5 (75.8–91.2)	

Se	100 (96.7–100)	98.9 (96.2–99.8)	100 (96.7–100)	Mainar-Jaime et al. (2005) [23]
Sp	86.4 (79.1–91.9)	100 (97.1–100)	94.4 (88.8–97.7)	

Âse	90.6 (81.6–98)	97.4 (93.2–99.9)	94.5 (89–98.3)	Mean Se and Sp
ÂSp	86.4 (71–99)	96.9 (92.5–99.8)	89 (82.3–94.4)	

**Table 2:** Cross tabulation of the three tests' results.

	CFT pos.		CFT neg.		Total
			I-ELISA		
	I-ELISA pos.	I-ELISA neg.	Pos.	neg.	
RBT pos.	2	0	2	1	5
RBT neg.	0	0	4	269	273

Total	2	0	6	270	278

**Table 3:** Observed estimate of sensitivities and specificities.

Test	Parameter	Posterior estimation
RBT	Se	89.6 (95% PI: 79.9–95.8)
	Sp	84.5 (95% PI: 68–94.8)

I-ELISA	Se	96.8 (95% PI: 92.3–99.1)
	Sp	96.3 (95% PI: 91.7–98.8)

CFT	Se	94 (95% PI: 87.8–97.5)
	Sp	88.5 (95% PI: 81–93.8)

Prevalence		4 (95% PI: 0.8–11.45)

rhoD		0.22 (95% PI: −0.05–0.71)

rhoDc		0.176 (95% PI: −0.082–0.64)

95% PI = 95% probability interval.
